# Solution Structure of LC4 Transmembrane Segment of CCR5

**DOI:** 10.1371/journal.pone.0020452

**Published:** 2011-05-27

**Authors:** Kazuhide Miyamoto, Kayo Togiya

**Affiliations:** 1 Department of Pharmaceutical Health Care, Faculty of Pharmaceutical Sciences, Himeji Dokkyo University, Himeji, Hyogo, Japan; 2 15-203 Hirano-machi, Himeji, Hyogo, Japan; Beckman Research Institute of the City of Hope, United States of America

## Abstract

CC-chemokine receptor 5 (CCR5) is a specific co-receptor allowing the entry of human immunodeficiency virus type 1 (HIV-1). The LC4 region in CCR5 is required for HIV-1 entry into the cells. In this study, the solution structure of LC4 in SDS micelles was elucidated by using standard 1H two-dimensional NMR spectroscopy, circular dichroism, and fluorescdence quenching. The LC4 structure adopts two helical structures, whereas the C-terminal part remains unstructured. The positions in which LC4 binds to the HIV-1 inhibitory peptide LC5 were determined by docking calculations in addition to NMR data. The poses showed the importance of the hydrophobic interface of the assembled structures. The solution structure of LC4 elucidated in the present work provides a structural basis for further studies on the HIV-1 inhibitory function of the LC4 region.

## Introduction

CC-chemokine receptor 5 (CCR5) is a member of the G-protein-coupled receptor superfamily and is comprised of seven transmembrane segments [1]. The N-terminus of the extracellular region is associated with binding of the chemokines, while the C-terminus of the intracellular region serves as a binding site for β-arrestin [2]. Furthermore, CCR5 and CXCR4 are specific co-receptors for human immunodeficiency virus type 1 (HIV-1) entry, which is the causative agent for AIDS [3], [4]. To gain entry into cells, HIV-1 requires a CD4 receptor and co-receptors such as CCR5 and CXCR4 [5], [6]. Blocking HIV-1 entry into the cell naturally leads to the inhibition of its infection and replication [7].

Recently, a novel synthetic LC5 peptide (^222^LRCRNEKKRHRAVRLIFTI^240^) that inhibits HIV-1 infection of MT-4 cells was reported [8]. It is suggested that the LC5 peptide interacts with the LC4 region (^157^VFASLPGIIFTRSQKEGL^174^) corresponding to the fourth transmembrane segment of CCR5 [8]. Gly163 in the LC4 region plays an important role in the formation of the complex between the gp120 envelope glycoprotein of HIV-1 and sCD4, and its mutation results in reduced susceptibility to HIV-1 [9]. LC4 is an attractive target for the inhibition of HIV-1 infection. The three-dimensional structure of the LC5 peptide was determined by using NMR methods in our previous study [10]. The peptide possesses an α-helical structure in the C-terminal region, and there is a hydrophobic cluster on the surface of the peptide. It is thought that the hydrophobic cluster contributes to binding with the LC4 region [10]. There is a growing interest in characterizing the structural conformation of the LC4 region. However, detailed information about the solution conformation of the LC4 region in the membrane environment at an atomic level and the mode of interaction with the membrane is unclear. Knowledge of the solution conformation of LC4 in the membrane is crucial for understanding the functional mechanism of the LC4 region.

The micellar environment of sodium dodecyl sulfate (SDS) micelles has been utilized to establish a reasonable model for the conformation of KcsA potassium channels in the membranes [11]. SDS micelles produce a membrane-mimetic environment allowing the structural study of the peptide LC5 [10] and Slc11a1 [12] in the membrane. Moreover, the membrane-mimetic environment of SDS micelles facilitates the characterization of the structural conformation of the transmembrane segments of membrane proteins [13].

Thus, in this study, we investigated the solution conformation of the LC4 region in the membrane-mimicking environment of SDS micelles using ^1^H-NMR spectroscopy, circular dichroism, and fluorescence quenching. In addition, the possible binding sites between the LC4 region and the LC5 peptide, which inhibits HIV-1 infection, were determined using docking calculations. This is the first conformational study of LC4 in the micellar environment.

## Materials and Methods

### Peptide synthesis

The LC4 peptide (^157^VFASLPGIIFTRSQKEGL^174^) corresponding to the LC4 region was synthesized with N-acetylated and C-amidated termini. Chemicals for peptide assembly, including amide resin, were obtained as SynProPep products from Shimadzu Corp. (Kyoto, Japan). After cleavage with trifluoroacetic acid, the peptide was purified on a reversed-phase HPLC using a Shim-pack C18 column (Shimadzu Corp.). The peptide purity was greater than 98%, and its molecular mass was assessed by MALDI-TOF MS on a Shimadzu AXIMA-TOF^2^.

### Circular dichroism (CD) spectroscopy

Far-UV CD spectra were recorded on a JASCO J-805 spectropolarimeter after calibration using d-camphor-10-sulfonate. The sample was dissolved in a buffer solution (80 mM phosphate buffer) or SDS solution (80 mM phosphate buffer, 10 mM SDS). A 0.1 mm path length quartz cell was used for 50 µM sample solution. Spectra at room temperature were acquired from 190 to 250 nm with a scanning speed of 50 nm/min, a response time of 4.0 s, and a bandwidth of 1 nm. Each CD spectrum was the average of four scans. After subtraction of the solvent spectrum, the CD data were obtained by converting from CD signal into mean residue molar ellipticity.

### Fluorescence

The fluorescence emission spectra were examined using a RF5300PC spectrofluorometer (Shimadzu Corp.). The sample was dissolved in a buffer solution or SDS solution, as described for the CD measurements. A 3×3 mm quartz cuvette was used for 50 µM sample solution. Fluorescence quenching of phenylalanine (Phe) was performed with 5–85 mM acrylamide of an extrinsic quencher, as described previously [10]. Phe was excited at 257 nm and its fluorescence emission at room temperature was acquired from 270 to 450 nm. After subtraction of the solvent spectrum, the fluorescence quenching data were used to calculate the Stern–Volmer constants (K_sv_) as follows [14], [15]:
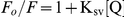
Where, F_o_ and F are the fluorescence intensities in the absence and presence of the quencher, respectively, and [Q] is the quencher concentration.

### NMR spectroscopy

For the NMR sample, the LC4 peptide (2 mM) was dissolved in^ 1^H_2_O/^2^H_2_O (9∶1) 80 mM phosphate buffer (pH 4.5) containing 200 mM sodium dodecyl-d_25_ sulfate (d_25_-SDS) (Sigma-Aldrich). NMR measurements were made at 20°C on a Bruker AVANCE 500 MHz. Two-dimensional NOESY spectra were recorded with 80, 100, 120, 150, and 200 ms mixing times [16]. Different mixing times were used to evaluate the linearity of the NOE build-up, as described previously [10]. The clean-TOCSY spectrum was recorded with a modified MLEV-17 spin-lock sequence where short delay times (Δ) of 40 µs were inserted into a conventional MLEV-17 sequence, i.e., 90°–Δ–180°–Δ–90° (mixing time, 80 ms) [17], [18]. The DQF-COSY spectrum was recorded to produce the resonance assignments [19]. In all the experiments, water suppression was achieved by using a WATERGATE pulse sequence [20]. The spectra were processed with the program NMRPipe [21] and the program NMRView [22] was used for optimal visualization and spectral analysis. The chemical shifts were referenced to the sodium salt of 3-(trimethylsilyl)-propionate-2,2,3,3-d_4_.

### Structure calculation

A peak list for the NOESY spectrum with a mixing time of 150 ms was generated by the peak picking and integration functions of NMRView [22]. Automated NOE cross-peak assignments and structure calculations with torsion angle dynamics were performed using the software package CYANA 2.1 [23]. Structure calculations were started from 100 randomized conformers, and the standard CYANA-simulated annealing protocol was used with 15,000 torsion angle dynamics steps per conformer. The 20 conformers with the lowest final CYANA target function values were validated using the program PROCHECK-NMR [24]. The Connolly surfaces of the structures were simulated using the program Discovery Studio 2.1 (Accelrys Software Inc.). The program MOLMOL [25] was used to analyze the resulting 20 conformers.

The atomic coordinates (code 2RRS) have been deposited in the Protein Data Bank, Research Collaboratory for Structural Bioinformatics.

### ZDOCK calculations

The docking calculation was performed using ZDOCK 3.0.1 [26]. ZDOCK is an initial-stage rigid-body molecular docking algorithm that uses a fast Fourier transform (FFT) algorithm to improve performance for searching in translational space. The precision of ZDOCK in predicting the docking structure has been proved in the Critical Assessment of Prediction of Interactions (CAPRI) Challenge [26]. All of the available structures from NMR were used to calculate the docking poses and the structures obtained were subjected to energy minimization using the Smart Minimizer algorithm (Max steps 200, RMS gradient 0.01) in the program Discovery Studio 2.1. The resulting top 5 conformers with the lowest energy were used as appropriate candidates.

## Results

### Secondary structure of LC4

**Figure 1:**
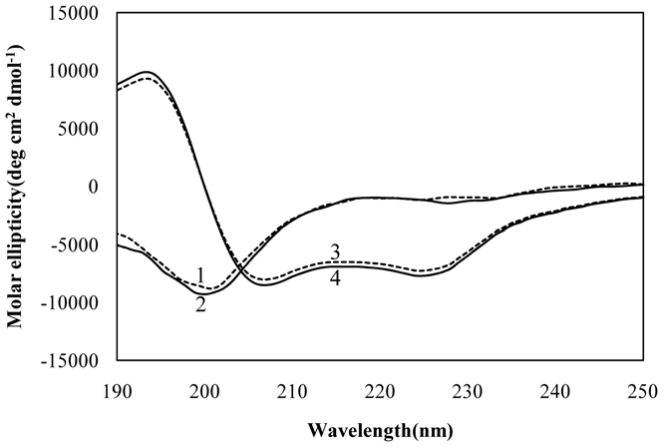
CD spectra of LC4. (1) 50 µM LC4 in phosphate buffer at pH 7.4; (2) as in (1) but at pH 4.5; (3) 50 µM LC4 in SDS micelles at pH 7.4; (4) as in (3) but at pH 4.5. The experiments were carried out at room temperature in 80 mM phosphate buffer or 80 mM phosphate buffer containing 10 mM SDS.

To gain information on the secondary structure of LC4, CD spectra of LC4 were recorded at pH 4.5 and 7.4 in an aqueous environment and in SDS micelles. As shown in Figure 1, the CD spectra of LC4 were little affected by the variation in pH in both the aqueous environment and SDS micelles. The CD spectra in phosphate buffers showed a single broad negative ellipticity centered at approximately 200 nm, indicative of a random-coil conformation. In contrast, in SDS micelles, a helical conformation was induced with spectral minima occurring at approximately 225 nm (n-π* transitions) and 207 nm (π-π* transitions) along with a strong positive ellipticity at approximately 195 nm characteristic of a typical helical structure [27].

### Insertion of LC4 into SDS micelles

**Figure 2:**
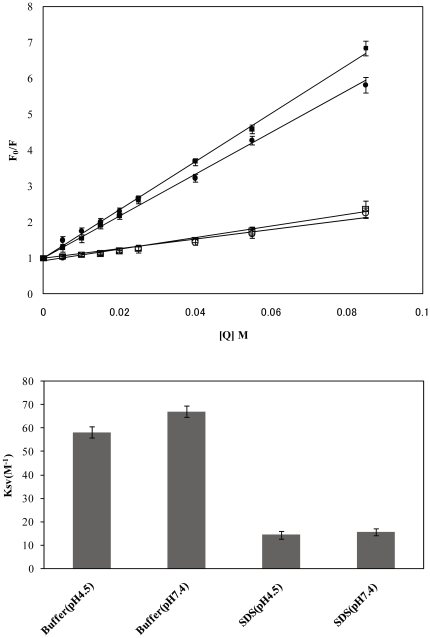
Insertion of LC4 into SDS micelles monitored by acrylamide quenching of LC4 fluorescence. (A) Stern–Volmer plot. (•), in phosphate buffer at pH 4.5; (▪), in phosphate buffer at pH 7.4; (○), in SDS micelles at pH 4.5; (□), in SDS micelles at pH 7.4. Peptide-coupled SDS micelles were preincubated for 15 min before measurements. [Q] is the quencher concentration (5–85 mM). The SDS micelles concentration was 10 mM, and the peptide concentration was 50 µM. (B) Stern–Volmer constants calculated from the acrylamide quenching experiments in (A). Each error bar shows the SEM from six measurements.

To characterize the degree of insertion of LC4 into SDS micelles, its solvent accessibility was evaluated by the fluorescence quenching of Phe with acrylamide. Stern–Volmer plots (Figure 2A) provided information on its solvent accessibility. The K_sv_ values in phosphate buffers at pH 4.5 and pH 7.4 are approximately 58.3 M^−1^and 67.1 M^−1^, respectively, while in the presence of SDS micelles, the K_sv_ values are approximately 14.5 M^−1^ and 15.6 M^−1^, respectively. The smaller K_sv_ values in SDS micelles indicate that the side chain of Phe is buried in the SDS micelles (Figure 2B). The aromatic rings of the residual Phe of LC4 are embedded into SDS micelles, irrespective of the pH of the solution.

### Resonance assignments

**Figure 3:**
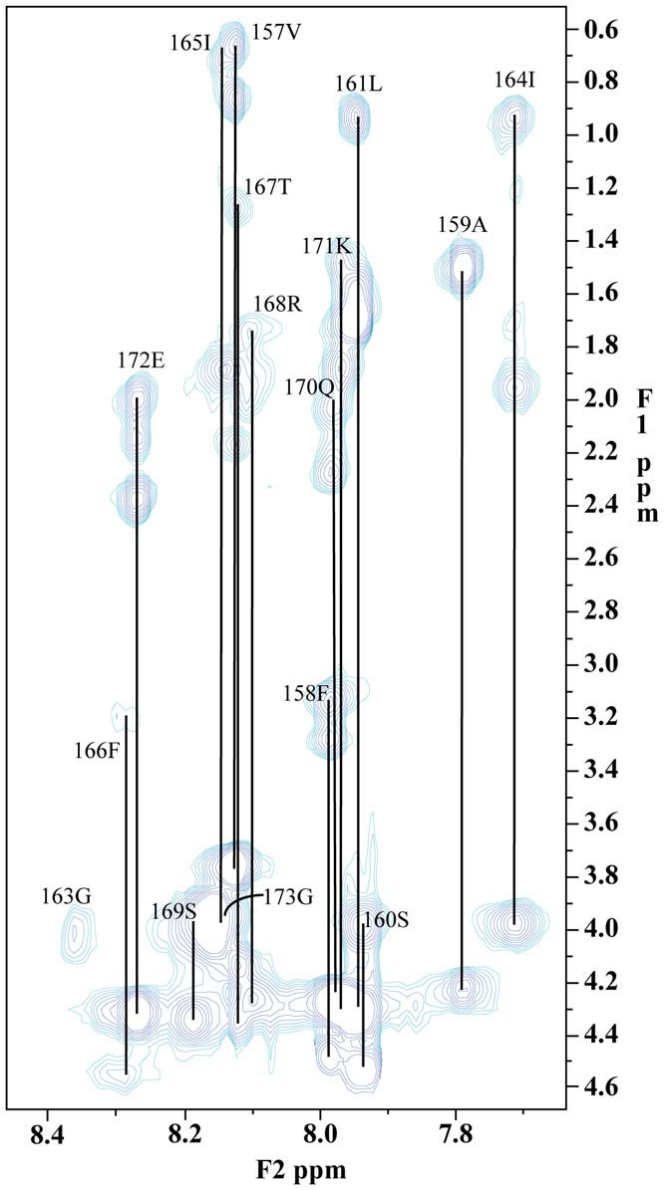
Two-dimensional clean-TOCSY spectrum (mixing time, 80 ms) of LC4 in d_25_-SDS micelles. The assignment of each residue is marked on the spectrum. The concentration of the LC4 peptide is 2 mM. The spectrum was recorded at 20°C in 80 mM phosphate buffer (pH 4.5) containing 200 mM d_25_-SDS micelles.

Two-dimensional ^1^H-NMR spectra of LC4 were obtained in SDS micelles at pH 4.5, because the exchange rates of amide protons at pH 4.5 are lower than those at pH 7.4 [28], [29], and the conformation of LC4 is not affected by pH, as indicated by the CD spectra. Assignment of the proton spectra was accomplished by standard sequential assignment techniques utilizing the identification of spin systems and NOE connectivities (Figure 3). The backbone resonance assignments were complete, with the exception of the amide group of Leu174. The signal of the amide proton of Leu174 was not observed in clean-TOCSY and NOESY spectra. With regard to the signals of the side-chain of Leu174, although they were very weak due to line broadening, their assignments were made by interactive interpretation of DQF-COSY, clean-TOCSY, and NOESY spectra. The proline side-chain resonances were assigned from the clean-TOCSY cross-peaks between H^δ^ protons and their respective H^γ^, H^β^, and H^α^ protons.

### Estimation of the secondary structure from chemical shifts and NOE connectivities

**Figure 4:**
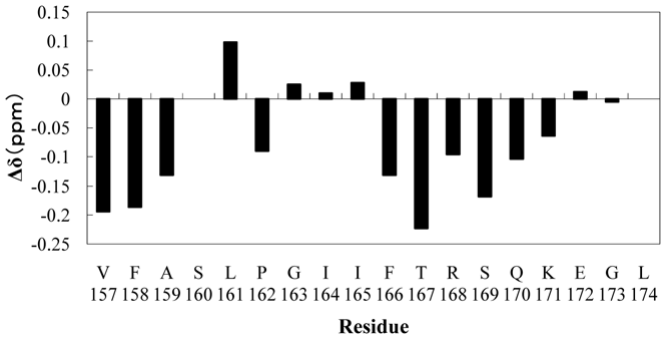
Chemical shift differences of H^α^ between the experimental values and random coil values. The experimental values were observed as the H^α^ chemical shift for 2 mM LC4 in the presence of 200 mM d_25_-SDS micelles. The random coil values were obtained from Chemical Shift Index [30]. The negative Δδ values showed the presence of the helical conformation.

**Figure 5:**
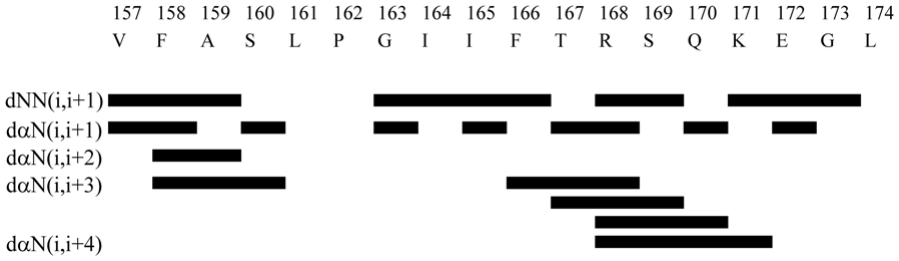
Patterns of sequential and medium range NOE cross-peaks of LC4 in SDS micelles. The NOEs for 2 mM LC4 were derived from the NOESY spectrum with a mixing time of 150 ms in the presence of 200 mM d_25_-SDS micelles. The NOE patterns are used to characterize the region of the helical structure [28].

The differences in the H^α^ chemical shift (Δδ in ppm) between the observed and the random coil values are shown in Figure 4. Negative Δδ values were observed at two regions, from V157 to A159 and from F166 to K171. A series of upfield H^α^ chemical shifts associated with random coil values showed the presence of an α-helical structure [30]. Thus, LC4 is considered to assume an α-helical conformation in these regions. Analysis of the NOESY spectrum provided the sequential and the medium range NOEs (|i–j| < 5) as summarized in Figure 5. The presence of dαN(i,i+3) and dαN(i,i+4) NOEs suggests an α-helical conformation; if dαN(i,i+2) NOEs are also observed, its region assumes a 3_10_-helical conformation [28]. Figure 5 shows that LC4 assumes an α-helical conformation from F166 to K171, as suggested by the H^α^ chemical shift. In addition, from F158 to S160, characteristic dαN(i,i+2) NOEs were also observed, and thus this region adopts a 3_10_-helical conformation.

### NMR structure

**Figure 6:**
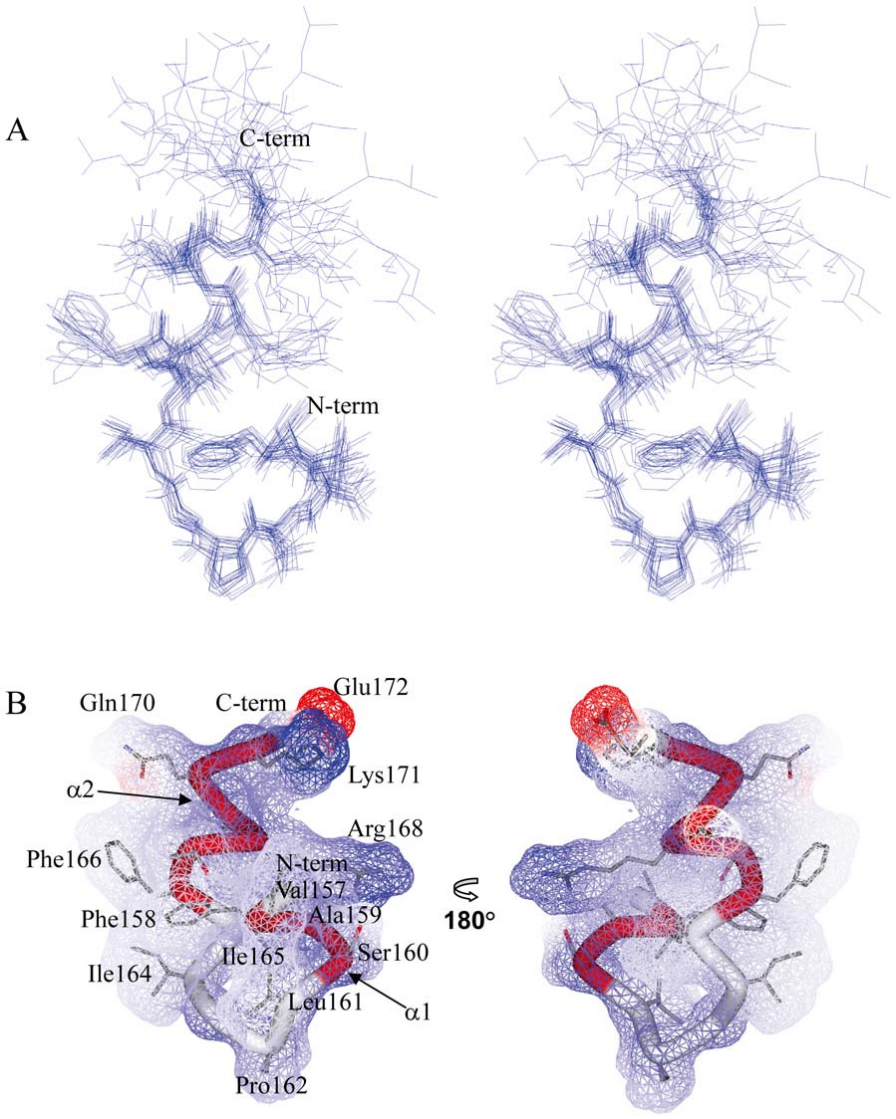
Overall structure of LC4 in SDS micelles at pH 4.5 by ^1^H-NMR. (A) Stereoview illustrating a trace of the backbone atoms for the ensemble of the 20 lowest energy structures, showing the heavy atoms of the side chains (residues Val157-Leu174). The structures in the well-ordered region (residues Val157-Glu172) was superimposed over the backbone atoms. (B) Surface representation and ribbon diagram of LC4 showing the side chains (residues Val157-Glu172). The helical regions (α1 and α2) are shown in red.

The three-dimensional structure of LC4 was determined using the torsion angle dynamics of the program CYANA [23]. A best fit superposition of an ensemble of the 20 lowest energy structures of LC4 in SDS micelles at pH 4.5 is shown in Figure 6A. The statistics of the structure as well as the distance restraints used for the program CYANA are summarized in Table 1. The 20 lowest energy structures in the well-ordered region (Val157–Glu172) are superimposed over the backbone (N, C^α^, C') atoms and the non-hydrogen atoms, with rms deviations of 0.52 Å and 1.17 Å, respectively. The C-terminal region of residues Gly173–Leu174 is not well ordered. The quality of the structures was checked using the program PROCHECK-NMR [24]. In a Ramachandran plot, 87.1% of the non-glycine residues were located within the most favored regions as shown in Table 1. The NMR results show that LC4 possesses two helical regions (α1 helix: Phe158–Ser160; α2 helix: Phe166–Lys171) in SDS micelles (Figure 6B). α1 and α2 adopt 3_10_-helical and α-helical structures, respectively, as indicated by the analysis of NOEs connectivities. Pro162 exists around the end of α1. It adopts a trans conformation, which was confirmed by the NOEs between the H^α^(i-1) and H^δ^(i) protons [28]. The residue Pro is referred to in a negative sense, it lacks an NH group, and often disrupts the formation of the secondary structure [31]. It appears that Pro162 links α1 and α2, and promotes the bend conformation between two helices. The Connolly surface of LC4 is shown in Figure 6B. The residues Val157, Phe158, Ala159, Leu161, Pro162, Ile164, Ile165, and Phe166 contribute to the formation of the hydrophobic cluster, which stabilizes the ordered structure of LC4 bound to micelles. Arg168, Lys171, and Glu172 gather at the molecular surface, and thus the structure of LC4 exhibits an amphipathic character in the micellar environment.

**Table 1:** Summary of Structure Statistics of LC4 in SDS micelles^a^.

NOE upper distance restraints	
Total	196
Intra residual and sequential	110
Medium range and long rage (i - i + 2, i - i + 3, i - i + 4)	86
CYANA target function value	0.09Å^2^
Distance constraints violations	
Number > 0.1 Å	0
Maximum	0.04Å
PROCHECK Ramachandran plot analysis^b^	
Residues in favored regions	87.1%
Residues in additionally allowed regions	12.9%
Residues in generously allowed regions	0.0%
Residues in disallowed regions	0.0%
RMS deviation to the average coordinates^b^	
Backbone atoms	0.52Å
Heavy atoms	1.17Å

a Except for the number of constraints, average values given for the set of 20 conformers with the lowest CYANA target function value.

b The values were calculated for residues 157–172.

### Possible docking poses

**Figure 7:**
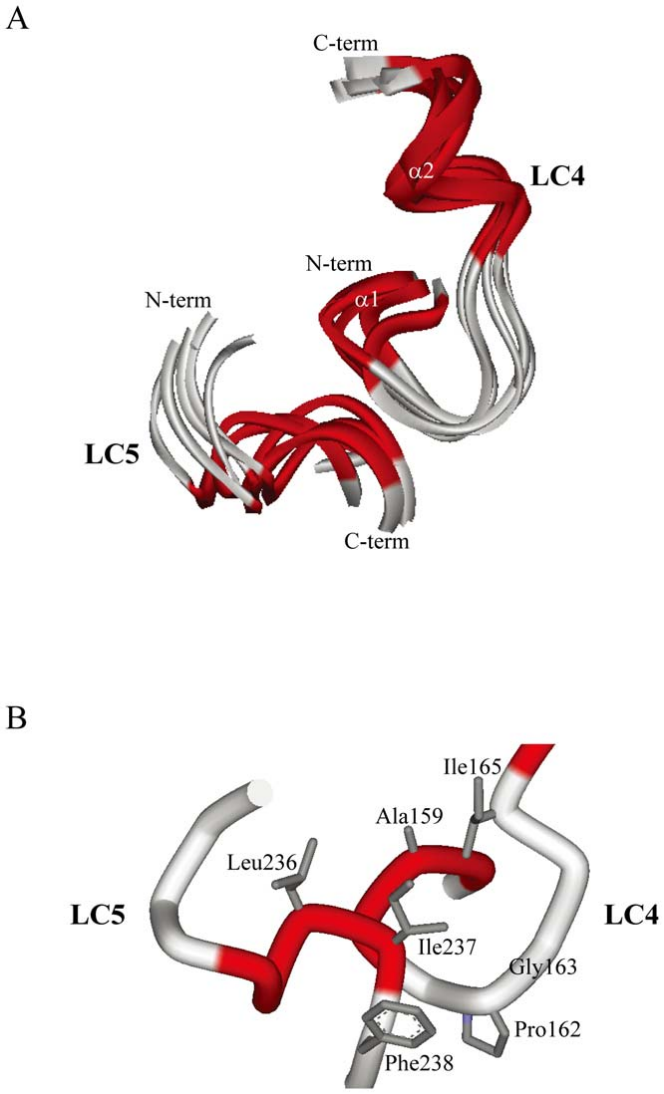
Possible docking positions of LC4 and LC5 calculated using the program ZDOCK. The NMR structure of LC5 was determined in the previous study [10]. The docking positions were calculated using the program ZDOCK and then the energy-minimization calculations were performed using the program Discovery Studio. (A) Ribbon diagrams of the 5 lowest energy structures (residues Val157–Glu172 of LC4; residues Lys229–Thr239 of LC5) (B) close-up view of the binding interface of the lowest energy structure, showing the heavy atoms of the side chains.

Docking of LC4 and LC5 was calculated using ZDOCK and Discovery Studio. The possible binding poses were obtained as 1,000 structures with the contacting residue Gly163, which is considered to be important in susceptibility to HIV-1. Figure 7A shows a best-fit superposition of the ensemble of the 5 lowest energy structures. The rms deviation is 1.15 Å for backbone (N, C^α^, C') atoms in all residues of the ordered region of LC4 and LC5. The α1 helical region of LC4 is spatially located in the vicinity of the helical region of LC5. Figure 7B shows the possible binding interface. Gly163, located next to residue Pro162 in LC4, is capable of contacting the Ile237 residue of LC5. In addition, in this position, the residues (Leu236, Ile237, and Phe238) of LC5 could interact with the residues (Ala159, Pro162, and Ile165) of LC4 which are included in the hydrophobic cluster. Therefore, the hydrophobic interactions between these residues preferentially contribute to the formation of the complex of LC4 and LC5.

## Discussion

The solution structure of LC4 in SDS micelles presented here provides an insight into its functional interaction with CCR5 at an atomic level. LC4 bound to micelles clearly shows two helical regions (α1 and α2) with a flexible C-terminal region of the peptide. The structure possesses a hydrophobic cluster and several charged residues preferentially gather on the molecular surface (Figure 6B).

The CD spectra showed that LC4 assumes a random-coil conformation in an aqueous environment, but a helical conformation in SDS micelles. Furthermore, acrylamide quenching fluorescence showed that LC4 is inserted into SDS micelles through Phe158 and/or Phe166. Both of these Phe residues contribute to the formation of a hydrophobic cluster that stabilizes the LC4 structure (Figure 6B), suggesting that LC4 is embedded into micelles through these two Phe residues. The hydrophobic cluster of the N-terminal region that includes these residues might be inserted into the micelles. In the favored model of the transmembrane region of CCR5, it is considered that residues Val157-Arg168 of LC4 are inserted into the membrane and residues Ser169-Leu174 exist in the extracellular region [8]. The hydrophobic cluster of LC4 is formed by the residues for the insertion into the membrane. The conformation of LC4 and the degree of insertion of Phe into the micelles were hardly affected by a variation in pH, as indicated by the CD spectra and acrylamide quenching fluorescence.

LC5 peptide inhibits HIV-1 infection of MT-4 cells, and its three-dimensional structure determined by NMR methods was reported in our previous study [10]. The docking calculations of LC4 with LC5 were performed using the program ZDOCK. The possible poses were obtained and superimposed with the rms deviation of 1.15 Å for backbone (N, C^α^, C') atoms over the ordered regions. The LC5 structure possesses a hydrophobic cluster in the C-terminal region. The interaction between the hydrophobic clusters of LC4 and LC5 leads to the formation of a hydrophobic interface on the molecular surface. This might be an important key to the inhibitory function of LC5, when LC4 and LC5 interact with each other. The LC5 structure adopts a stable α-helical conformation in the micelles as a model membrane, irrespective of variation in the pH of the solution. However, the replacement of Phe238 with Ala results in a dramatic conformational change of LC5, suggesting that the structure could possess two helical regions [10]. Phe238 is included in the hydrophobic cluster, and thus its mutation could affect the interaction and formation for the hydrophobic clusters of LC4 and LC5. Therefore, it is tempting to speculate that Phe238 contributes to the inhibitory activity of LC5.

In conclusion, the present study provides the first structural report of the LC4 region of CCR5 in SDS micelles. We also demonstrated that LC4 is embedded into the model membrane micellar environment through Phe residues. Moreover, the possible docking poses calculated by using the NMR structures of the peptides LC4 and LC5 show that their hydrophobic clusters interact with each other. Further studies are required to shed light on the role of the LC4 region of CCR5 in its HIV-1 inhibitory function.
